# Archives of Drug Information—A New Approach to Publishing the Results of Drug Studies

**DOI:** 10.1111/j.1753-5174.2007.00001.x

**Published:** 2008-07

**Authors:** C Michael Stein

**Affiliations:** Vanderbilt University Medical School542 RRB, Nashville, TN, USA

## Abstract

Current scientific publishing uses a selective, slow, and adversarial editorial process to publish a minority of papers submitted, and thus maximize a journal's impact factor, a major measure of success. However, the results of many pharmaceutical industry studies are deemed low priority and are therefore difficult or impossible to publish in scientific journals. Society is poorly served because access to the results of these studies is in the public interest. *Archives of Drug Information* is an independent, online only journal that will publish papers that are free of bias and that provide new information about a drug. Articles that would not usually be published by traditional journals, for example articles reporting negative studies, studies reporting animal toxicity or Phase I human studies, or routine pharmacokinetic or drug interaction studies are welcome. The editorial process will be rapid and user friendly. The contents of the journal will be available freely to all. *Archives of Drug Information* will allow the pharmaceutical industry to publish the results of studies and make freely accessible to the public the results of research studies that would otherwise remain unavailable “on-file.” Making the results of studies available to the public is not only ethical and good scientific citizenship, but ultimately, also good business.

Scientific publishing focuses on the selective publication of the highest quality articles appropriate for a particular journal. Acceptance rates of around 30 percent are common, and priority is accorded to papers that are hypothesis-based and of general interest within a particular scientific area. The success of a journal is often judged by its impact factor, thus there is a strong motivation to selectively publish those articles that are likely to be cited widely. The current submission process is an iterative and adversarial one with reviewers trying to find reasons why a paper should not be published. Many methodologically sound papers are rejected based on the priority accorded them by the reviewers and the editor. This model suits the academic world, but it is poorly suited to industry.

## Current Scientific Publishing and the Pharmaceutical Industry

The needs of the pharmaceutical industry are not met by scientific journals. Much of the data produced by industry are descriptive rather than hypothesis driven, and much of this information is of interest to only a small number of individuals. For example, negative studies, or a pharmacokinetic study of compound that does not yet have a name (XYZ 4567) and has a high likelihood of being withdrawn from development in subsequent years, are not a high priority for scientific journals.

The protracted and iterative editorial process, while suited to an academic environment, is poorly suited to early drug development studies in an industry where the emphasis is on presenting the data rather than on producing a well-argued scientific package. Thus, one of the reasons so many industry studies are never published is that much of the work would not be accepted by scientific journals.

## Scientific Publishing and Society

A recent review points out the challenges to the registration of clinical trials, and draws attention to an even greater problem—the results of many clinical trials are never made public [1]. Society is poorly served because access to the results of unpublished studies is in the public interest. There are several reasons for this. First, it will prevent scientists in drug development from repeating mistakes or following the same blind alleys others have explored. Subjecting humans to studies when risks, or lack of efficacy, have previously been recognized, but not made public, is ethically unacceptable [2]. Second, making the results of studies freely available allows the scientific world access to information, and thus the ability to assess risks and benefits independently. The pharmaceutical industry has been criticized for not publishing the results or studies performed in the development of a drug, particularly when adverse effects were reported later. Transparency is the best protection against later allegations that data were deliberately hidden from the public.

## Making the Results of Studies Available

There has been a public demand that the results of all studies should be made available and placed in the public domain. Several pharmaceutical companies and organizations have created databases for clinical trial results. Many have the following disadvantages: they are difficult to search and navigate; the quantity of information provided varies, but is often only a summary; it is unclear who the authors of the report are; it is unclear how the database is curated; there is no external peer review; and there is a perceived lack of independence. *Archives of Drug Information* seeks to remedy the obstacles to publication of research studies about drugs.

## About *Archives of Drug Information*

*Archives of Drug Information* is an independent, online-only, peer-reviewed journal that provides high quality, rapid, efficient publication of original unbiased scientific articles about drugs. It makes available to the public information that other journals may regard as being of low priority for publication and of archival interest, and provides free access to citable and searchable information. *Archives of Drug Information* does not charge readers but authors pay a manuscript fee. The contrasts between *Archives of Drug Information* and current scientific literatures are summarized in Table 1.

**Table 1:** Current Scientific Journals and Archives of Drug Information—differences and similarities

Current journals	*Archives of Drug Information*
Only publish the top 1/3 of papers	Publishes suitable papers
Peer review that is adversarial	Peer review that aims to avoid bias, ensure quality and improve an article for readers
Slow, painful and subjective editorial process	Rapid, painless and transparent editorial process
Publishes hypothesis-based work	Publishes hypothesis-based and descriptive information
Publishes work in a narrow area	Publishes a broad range of work about drugs—chemistry, cell, animal, human studies
Publishes work that is of wide interest	Publishes work that may be of limited interest
Emphasis in papers is on the Introduction that sets up the scientific rationale, and on the Discussion that demonstrates scientific worth	Emphasis is on the data and how they were obtained, i.e. Methods and Results
Space is limited—selective results are presented	Space is not limited
Impact factor affects editorial policy	Impact factor is unimportant
Provides access to a small amount of information	Provides access to a large amount of information
Paper and electronic	Electronic only
Lag time 2–4 months	Lag time weeks
Publishes monthly	Publishes when MS processed
Subscriber pays	Author pays
Subscriber reads or scans the journal	User searches for relevant information
Access limited to subscribers	Open access

## What Does *Archives of Drug Information* Publish?

The *Archives* publishes papers presenting original research about drugs. Papers that would be of low priority for traditional scientific journals but are the routine work of drug development, for example early drug development studies, routine pharmacokinetic or drug-drug interaction studies, dose-finding studies, animal toxicity studies, pilot studies, and negative clinical studies are welcome. A broad range of work including cellular, animal and human studies are of interest, but we do not publish opinion pieces, meta-analyses or review articles.

## Criteria for Publication in *Archives of Drug Information*

To be published, a paper must fulfill the following criteria:

- Present original research about a drug or compound that has not been published elsewhere.
- Studies involving humans and animals must be performed ethically and approved by the authors' institutional review board or its equivalent.
- Studies are of high quality with the methods and results fully described. Weaknesses and limitations are acknowledged.
- Information is free of bias.

Given the emphasis of the *Archives* on data, the emphasis of papers will be on the Methods and Results sections. The Introduction can be concise; a few sentences will suffice to state the objectives of the study. The Discussion will be brief without speculation or opinion and should highlight the limitations of the study. A Conclusion is not required. The focus of *Archives of Drug Information* is on presenting data, not opinion about what the data might mean.

## Moving from “On-file” to “Online”

Making the results of studies freely available is not only ethical and good scientific citizenship, but ultimately, also good business. *Archives of Drug Information* will allow the pharmaceutical industry and others to publish the results of studies and make freely accessible to the public the results of research studies that would otherwise remain unavailable “on-file.”
